# Reliability and minimal detectable change of the ‘Imperial Spine’ marker set for the evaluation of spinal and lower limb kinematics in adults

**DOI:** 10.1186/s13104-020-05295-9

**Published:** 2020-10-22

**Authors:** J. A. Deane, E. Papi, A. T. M. Phillips, A. H. McGregor

**Affiliations:** 1 Sackler MSK LAB, Sir Michael Uren Hub, Imperial College London, White City Campus, 86 Wood Lane, London, W12 0BZ UK; 2 Structural Biomechanics, Department of Civil and Environmental Engineering, Imperial College London, London, UK

**Keywords:** Marker set, Kinematics, Spine, Low back pain, Gait, Sit to stand, Minimal detectable change, Reliability, Three dimensional motion capture, Motion technology

## Abstract

**Objectives:**

As a step towards the comprehensive evaluation of movement in patients with low back pain, the aim of this study is to design a marker set (three rigid segment spine, pelvic and lower limb model) and evaluate the reliability and minimal detectable change (MDC) of this marker set in healthy adults during gait and sit to stand (STS) tasks using three dimensional motion capture.

**Results:**

The ‘Imperial Spine’ marker set was used to assess relative peak angles during gait and STS tasks using the minimum recommended sample size (n = 10) for reliability studies with minimum Intraclass Correlation Coefficient (ICC) of 0.70, optimum ICC 0.90 and 9 trials replicated per subject per task. Intra- and inter-tester reliability between an experienced and inexperienced user was examined. ICC, mean, standard error (SEM), Bland Altman 95% limits of agreement (LOA) and MDC were computed.

ICC values demonstrated excellent intra- and inter-tester reliability in both tasks, particularly in the sagittal plane (majority ICCs > 0.80). SEM measurements were lower in gait (0.8–5.5°) than STS tasks (1°-12.6°) as were MDC values. LOA demonstrated good agreement. The ‘Imperial Spine’ marker set is reliable for use in healthy adults during functional tasks. Future evaluation in patients is required.

## Introduction

The ‘Imperial Spine’ marker set was developed to assess spinal and lower limb movement or kinematics during functional tasks using three dimensional motion capture (3DMC). To date, spinal movement has been examined in both healthy and patient populations using regional lumbar [1–3] or multiple spine segments [4–6]. Although, some consider the contribution of the spine, pelvis and lower limbs towards assessing spinal movement, few have analysed the absolute measures of measurement error and minimal detectable change (MDC). MDC describes the amount of change that is greater than the measurement error for each joint and plane of movement [7]. This permits kinematic data to be interpreted in a clinically meaningful manner, enabling the assessment of true differences.

Low back pain (LBP) is an extremely common symptom [8] associated with difficulties walking and sitting to standing (STS) [9]. Since current LBP management is at best, moderately effective [10], it is necessary to consider alternative therapeutic targets. Steps have been taken towards this through the examination of spine and lower limb segment motion in healthy adults using one and two rigid spinal models [7, 11]. However, spinal models with more than two rigid segments will be required in order to reliably characterise and interpret movement during activities that are important to LBP patients [12].

This preliminary study builds upon previous research through the development of a three rigid segmented spine, pelvic and bilateral lower limb marker set, the ‘Imperial Spine’. The objective of this study is to establish the reliability and MDC values relating to the ‘Imperial Spine’ in adults during gait and STS tasks using 3DMC as a step towards evaluation in LBP patients.

## Main text

### Methods

The sample size was determined and study design optimised using recommendations previously described ($\alpha =0.05, \beta =0.20)$ [13]. Healthy adults (4 males, 6 females) were recruited from University staff (mean age 30.8 (25.8–35.8) years, mean body mass index 23.4 (19.0–27.6) kg/m^2^). Strict criteria ensured that participants had no current or past history of LBP, spine or lower limb extremity trauma, neurological or musculoskeletal history that would affect task performance. Each participant provided written informed consent (REC Ref. 15IC2985).

### Reliability testing

Reliability of the ‘Imperial Spine’ marker set was evaluated using testers with and without prior clinical knowledge; tester 1(JD) (physiotherapist, 16 years clinical experience) and tester 2 (EP) (biomechanist, no prior clinical experience). Prior to subject testing each tester completed training including marker set familiarisation (30 min) and practical training (60 min) using standardised written instruction to reduce tester bias.

Each session comprised of 5 gait and 5 STS trials, 2 of which involved participant familiarisation. The gait task required unshod participants to walk at a comfortable speed over a level 6 m walkway at a self-selected pace. The STS task required participants to stand up from a backless chair with arms crossed, knees initially flexed to 90° and both feet assuming a ‘natural stance position’. Participants followed standardised verbal instruction.

Prior to the first session tester 1 (JD1) applied the marker set to participants using double-sided tape. On completion of the tasks, the marker set was systematically removed by tester 1 using alcohol swabs to remove signs of adhesive. An interval of 45 min was observed to ensure participant rest and to engage tester 1 in unrelated activities to minimise memory bias. During the second session the marker set was then re-applied and removed by tester 2 (EP) as described. Following the same interval, tester 1 repeated this sequence (JD2).

Testers were not permitted to observe each other or communicate during testing and were blinded to all kinematic outputs.

### The ‘Imperial Spine’ marker set and data processing

**Fig. 1:**
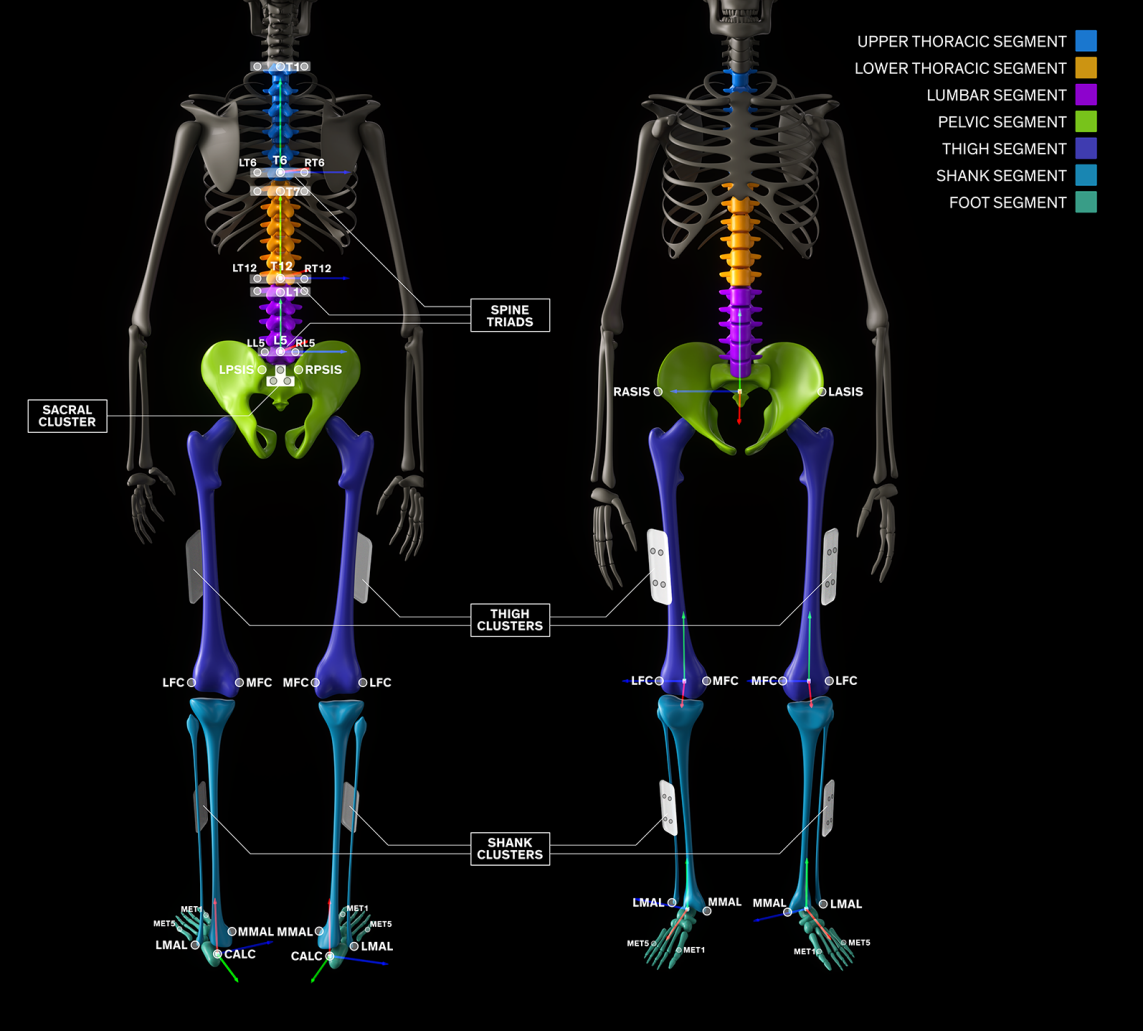
The ‘Imperial Spine’ marker set, segments and local anatomical frames. For all local anatomical frames, the + y axis (cephalad) is indicated in green, the + z axis (towards the right) in blue and the + x axis (perpendicular to both + y and + z axes) in red

The ‘Imperial spine’ was modelled in three segments according to easily identifiable anatomical landmarks; upper thoracic (T1-T6), lower thoracic (T7-T12) and lumbar (L1-L5). The upper thoracic (UT) segment was defined with its origin in T6, vertical axis from T6 to T1 (+ y) and horizontal axis through T6 (+ z to the right). The lower thoracic (LT) segment was defined with its origin in T12, vertical axis from T12 to T7 (+ y) and horizontal axis through T12 (+ z to the right). The lumbar (L) segment was defined with its origin in L5, vertical axis from L5 to L1 (+ y) and horizontal axis through L5 (+ z to the right) (Fig. 1). Pelvic, hip, thigh, shank and foot local co-ordinate systems were also defined and reconstructed from joint centres and easily identifiable anatomical landmarks on the pelvis and lower limb [14–16].

Anatomical frames of the pelvis, thigh and shank were referenced to the corresponding technical frames (constructed from technical clusters of markers) in the static calibration trial such that anatomical markers (ASIS, PSIS, MFC, LFC, LMAL, MMAL) (Fig. 1; Additional file 1: Table S1) could be removed prior to dynamic trial, permitting freedom of movement. All trials were recorded at 100 Hz using a 10-camera 3DMC system (Vicon Nexus (T160), Oxford Metrics Ltd., Oxford, UK) [17].

The onset and cessation of each task were determined using kinematics from each gait [18] and STS motion cycle [12, 19]. Each cycle was extracted (Vicon Nexus (T160), Oxford Metrics Ltd., Oxford, UK) and filtered using a Woltring cross-validity quantic spline routine [20]. The data was then normalised to 100% of each motion cycle (MATLAB, Mathworks, Natick, MA., U.S.A.). 3D Kinematics of each segment and joints were calculated using the Joint Coordinate System (JCS) convention [21] and computed using Bodybuilder and Vicon Nexus software (Oxford Metrics Ltd., Oxford U.K.). The average relative peak angles were then extrapolated.

### Statistical analysis

The normality of the data was confirmed using Q-Q plots and the Shapiro Wilks test (significance level *p* ≥ 0.05). Inter-tester and intra-tester ICCs (3, k) (2-way mixed model) and the 95% confidence intervals were derived. ICC values of 0.70 were considered acceptable, 0.75–1.00 excellent, 0.40–0.74 fair to good and ≤ 0.40 poor [22].

The mean peak joint angles (mean session one and two measurements), mean of the differences between measurements at session one and two (Mean Diff), the respective 95% confidence intervals (95% CI) for these differences, the standard deviation of the differences (SD Diff) and the 95% levels of agreement (95% LOA) were determined [23] in frontal, sagittal and transverse planes. The standard error of measurement (SEM) was calculated $(SEM = SD\;Diff \div \sqrt {2} )$ [24]. The minimal detectable change (MDC), which expresses the amount of joint angle change was also calculated $(MDC=1.96\times \sqrt{2}\times SEM)$ [25].

ICC statistical analysis was conducted using SPSS software (SPSS Statistics Version 22, IBM, Chicago, IL., U.S.A.). A critical level p < 0.05 was defined as significant. Mean, Mean Diff, 95% CI, SD Diff, 95% LOA, SEM and MDC calculations were computed using Microsoft Excel (Excel 2010, Microsoft Corporation, Redmond, WA., U.S.A.).

### Results

#### Gait task

Analysis of the mean peak joint angles for the spine and lower limbs demonstrated that 70% of intra-tester and 76% of inter-tester ICC scores were excellent (0.75–0.99). The remainder ranged between 0.60–0.74 (intra-tester) and 0.50–0.56 (inter-tester). Overall, ICC values were higher in the sagittal plane (for both intra- and inter-tester reliability), whilst those in the frontal and transverse planes were lower (Table 1). Kinematic waveforms reflect this agreement (Additional file 2: Figure S1, Additional file 3: Figure S2 and Additional file 4: Figure S3).

**Table 1:** Gait task

Kinematic angles (°) for Gait trial	Intra-tester									Inter-tester								
	ICC	95% CI	Mean	Mean diff	95% CI	SD diff	95% LOA	SEM	MDC	ICC	95% CI	Mean	Mean diff	95% CI	SD diff	95% LOA	SEM	MDC
Upper thoracic joint																		
Peak right/left side flexion	0.7	− 1.1	2.2	− 0.6	− 2.12–0.99	2.5	− 5.48–4.35	1.8	4.9	0.82	0.31–0.98	1.9	0	− 1.55–1.54	2.5	− 4.89–4.87	1.8	4.9
Peak internal/external rotation	0.95	0.81–0.99	6.1	-2.1	− 3.06–1.23	1.5	− 5.03–0.75	1.1	2.9	0.85	0.39–0.96	5.7	− 1.3	− 2.78–0.20	2.4	− 5.99–3.41	1.7	4.7
Peak flexion/extension	0.99	0.97–0.99	25.4	− 0.3	− 1.06–0.40	1.2	− 2.63–1.98	0.8	2.4	0.99	0.96–0.99	25.3	− 0.1	− 0.89–0.63	1.2	− 2.53–2.27	0.8	2.4
Lower thoracic joint																		
Peak right/left side flexion	0.6	0.77–0.89	1.4	− 1.63	− 2.96–− 0.30	2.15	− 5.84–2.58	1.5	4.2	0.55	− 1.72	0.6	− 0.2	− 2.00–1.71	3.0	− 6.02–5.72	2.1	5.9
Peak internal/external rotation	0.83	0.32–0.96	2.7	0.9	− 0.52–2.23	2.2	− 3.49–5.21	1.6	4.3	0.24	− 2.86	3	0.3	− 0.46–1.10	1.3	− 2.15–2.78	0.9	2.5
Peak flexion/extension	0.94	0.77–0.99	− 5	0.2	− 2.37–2.68	4.1	− 7.84–8.15	2.9	8	0.96	0.84–0.99	− 5.3	0.8	− 1.47–3.01	3.6	− 6.31–7.86	2.5	7.1
Lumbar																		
Peak right/left side flexion	0.74	− 0.99	6.7	1.3	− 0.55–3.10	2.9	− 4.50–7.04	2.1	5.7	0.52	− 1.83	6.9	0.9	− 2.15–4.00	5.0	− 8.80–10.64	3.5	9.8
Peak internal/external rotation	0.61	− 1.48	1.3	− 0.1	− 2.52–2.32	3.9	− 7.76–7.55	2.8	7.6	0.76	0.02–0.94	0.7	1.1	− 0.69–2.92	2.9	− 4.59–6.82	2.1	5.7
Peak flexion/extension	0.85	0.40–0.96	3.7	− 2.2	− 6.40–2.04	6.8	− 15.51–11.15	4.8	13.3	0.82	0.26–0.96	3.2	− 1.2	− 6.06–3.64	7.8	− 16.54 to14.13	5.5	15.3
Pelvic joint																		
Peak tilt	0.6	− 1.71	3.2	0.7	− 3.28–4.69	6.4	− 11.90–13.31	4.5	12.5	0.56	− 1.84	12.5	0.1	− 2.06–2.17	3.4	− 6.63–6.74	2.4	6.7
Peak rotation	0.93	0.68–0.98	10.2	− 0.9	− 2.49–0.74	2.6	− 5.98–4.24	1.8	5.1	0.82	0.19–0.96	10.9	− 1.7	− 4.11–0.67	3.9	− 9.28–5.84	2.8	7.6
Peak obliquity	0.6	− 1.79	4.5	− 0.7	− 2.93–1.59	3.7	− 7.82–6.48	2.6	7.3	0.5	− 2.18	4.1	− 0.6	− 3.09–1.83	4.0	− 8.41–7.14	2.8	7.8
Hip joint																		
Peak abduction/adduction	0.79	0.05–0.95	7.3	0.2	− 2.11–2.45	3.7	− 7.04–7.38	2.6	7.3	0.77	0.01–0.95	6.4	1.4	− 1.02–3.76	3.9	− 6.18–8.92	2.8	7.6
Peak internal/external rotation	0.9	0.56–0.98	8.7	0.8	− 3.23–4.87	6.5	− 11.98–13.63	4.6	12.7	0.87	0.41–0.97	6.8	3.4	− 0.82–7.65	6.8	− 9.97–16.8	4.8	13.3
Peak flexion/extension	0.79	0.69–0.95	30.4	0.7	− 3.92–5.41	7.5	− 14.00–15.49	5.3	14.7	0.78	0.28–0.95	29.1	− 0.7	− 5.27–3.92	7.4	− 15–13.86	5.2	14.5
Knee joint																		
Peak abduction/adduction	0.94	0.75–0.99	5.9	− 1.1	− 2.19–0.08	1.8	− 4.64–2.54	1.3	3.5	0.98	0.89–0.98	5.4	− 1.1	− 1.92–0.25	1.3	− 3.7–1.5	0.9	2.5
Peak internal/external rotation	0.91	0.59–0.98	− 7.2	− 0.8	− 5.48–3.96	7.5	− 15.57–13.96	5.3	14.7	0.91	0.62–0.98	− 6.2	− 1.4	− 5.46–2.67	6.6	− 14.3–11.5	4.7	12.9
Peak flexion/extension	0.93	0.69–0.98	66.6	0.1	− 0.98–1.21	1.2	− 3.34–3.57	0.8	2.4	0.92	0.62–0.98	67.2	− 0.9	− 2.12–0.36	2.0	− 4.79–3.02	1.4	3.9
Ankle joint																		
Peak abduction/adduction	0.92	0.68–0.98	12.5	− 0.8	− 3.55–2.02	4.5	− 9.57–8.04	3.2	8.8	0.92	0.67–0.98	12.5	0.1	− 2.06–2.17	3.4	− 6.63–6.74	2.4	6.7
Peak inversion/eversion	0.96	0.82–0.99	21.4	0.1	− 1.21–1.31	2.1	− 3.92–4.02	1.5	4.1	0.96	0.83–0.99	21.8	0.3	− 1.02–1.52	2.0	− 3.75–4.25	1.4	3.9
Peak dorsiflexion/plantarflexion	0.72	− 1.19	11.8	− 0.7	− 2.14–0.71	2.3	− 5.22–3.79	1.6	4.5	0.93	0.67–0.98	11.8	− 0.3	− 1.19–0.55	1.4	− 3.07–2.43	1.0	2.7

ICC (intraclass correlation coefficient), 95% CI (the 95% confidence interval for the ICC), Mean (average angle measured between tester 1 on 2 occasions (intra-tester) and tester 1 and 2 (inter-tester)), Mean Diff (represents the average of the differences between two measurements made by tester 1 on two occasions (intra-tester) and between tester 1 and 2 (inter-tester)) and the 95% CI for Mean Diff, SD Diff (the standard deviation of the differences), 95% LOA ( Bland and Altman 95% limits of agreement), SEM (standard error of measurement) and MDC (absolute minimal detectable change)

The SEM values were ≤ 5.3° and ≤ 5.5° for all intra- and inter-tester trials respectively, with 91% of values falling below 5°. The mean differences between sessions for all parameters were lower for intra-tester trials (≤ 0.9°, except 1.3° for peak lumbar abduction/adduction) than inter-tester trials (≤ 1.4°, except 3.4° for peak hip internal/external rotation). The MDC values ranged between 2.4 and 4.7° (intra-tester) and 2.4°–15.3° (inter-tester).

Bland Altman 95% limits of agreement for both intra-tester and inter-tester trials are outlined in Table 1.

### STS task

The mean peak joint angles for the spine and lower limbs demonstrated ICC ranges of − 0.82–0.98 (intra-tester) and − 0.52–0.97 (inter-tester). 76% of intra-tester and 52% of inter-tester ICC scores indicated excellent reliability (0.75–0.99). ICC values were higher in the sagittal plane; 0.83–0.98 (intra-tester) and 0.89–0.97 (inter-tester, except 0.52 at the ankle) and lower in the transverse and frontal planes (− 0.2 to 0.89) (Table 2). Kinematic waveforms reflect this agreement (Additional file 2: Figure S1, Additional file 3: Figure S2 and Additional file 4: Figure S3).

**Table 2:** STS task

Kinematic angles (°) for STS trial	Intra-tester									Inter-tester								
	ICC	95% CI	Mean	Mean diff	95% CI	SD diff	95% LOA	SEM	MDC	ICC	95% CI	Mean	Mean diff	95% CI	SD diff	95% LOA	SEM	MDC
Upper thoracic joint																		
Peak right/left Side Flexion	− 0.36	− 5.12	0.6	− 0.82	− 2.86–1.22	3.29	− 7.26–5.62	2.3	6.4	0.66	− 1.3	0.2	0.01	− 1.82–1.84	2.96	− 5.79–5.8	2.1	5.8
Peak internal/external rotation	0.84	0.35–0.96	2.7	− 0.48	− 1.40–0.44	1.48	− 3.30–2.43	1.0	2.9	− 0.2	− 4.51	2.6	− 0.25	− 1.73–1.24	2.40	− 4.95–4.45	1.7	4.7
Peak flexion/extension	0.98	0.93–0.99	20.8	− 0.77	− 1.87–0.33	1.77	− 4.25–2.71	1.3	3.5	0.97	0.91–0.99	20.5	− 0.19	− 1.38–1.00	1.92	− 3.96–3.58	1.4	3.8
Lower thoracic joint																		
Peak right/left side flexion	− 0.82	− 6.86	− 0.6	− 0.44	− 2.92–2.04	4.01	− 8.29–7.42	2.8	7.9	0.62	− 1.45	− 0.9	1.48	− 2.03–2.34	3.52	− 6.76–7.06	2.5	6.9
Peak internal/external rotation	0.36	− 2.42	2	0.69	− 0.66–2.44	2.18	− 3.59–4.97	1.5	4.3	0.6	− 1.5	1.9	− 0.2	− 0.36–2.10	1.99	− 3.02–4.76	1.4	3.9
Peak flexion/extension	0.9	0.62–0.98	− 1.7	0.47	− 1.72–2.65	3.53	− 6.45–7.38	2.5	6.9	0.92	0.66–0.98	− 1.4	− 0.28	− 1.98–1.43	2.75	− 5.66–5.10	1.9	5.4
Lumbar joint																		
Peak right/left side flexion	0.05	− 3.57	2.1	1.48	− 1.01–3.97	4.02	− 6.40–9.36	2.8	7.9	0.42	− 2.2	3.4	− 1.27	− 5.07–2.53	6.13	− 13.29–10.74	4.3	12
Peak internal/external rotation	0.79	0.15–0.95	− 0.6	− 0.2	− 1.96–1.56	2.83	− 5.76–5.35	2.0	5.5	0.31	− 2.53	− 0.4	− 0.61	− 2.82–1.59	3.56	− 7.59–6.37	2.5	7
Peak flexion/extension	0.82	0.29–0.96	13.3	− 0.28	− 5.76–5.20	8.84	− 17.60–17.05	6.3	17.3	0.89	0.58–0.97	11.2	3.9	− 0.19–7.98	6.58	− 25.81	4.7	12.9
Pelvic joint																		
Peak tilt	0.82	0.19–0.96	24.6	2.12	− 4.09–8.26	9.91	− 17.30–21.54	7.0	19.4	0.91	0.59–0.98	25.7	− 1.84	− 6.35–2.67	7.28	− 16.10–12.43	5.1	14.3
Peak rotation	− 0.29	− 5.45	17.8	− 2.38	− 13.42–8.67	17.82	− 37.30–32.55	12.6	34.9	0.2	− 3.38	20.1	− 5.88	− 13.97–2.21	13.05	− 31.47–19.71	9.2	25.6
Peak obliquity	0.88	0.48–0.97	1.3	-0.14	-1.33–1.05	1.92	3.63–-3.91	1.4	3.8	0.14	-3.61	1.9	-1.99	-5.24–1.25	5.23	-12.25–8.27	3.7	10.3
Hip joint																		
Peak abduction/adduction	0.9	0.10–0.50	0.4	1.2	− 0.92–3.39	3.50	− 5.58–8.05	2.5	6.9	− 0.52	− 6.35	0.8	0.17	− 4.39–4.74	7.40	− 14.27–14.61	5.2	14.5
Peak internal/external rotation	0.95	0.10–0.80	3.6	2.1	− 0.53–4.60	4.20	− 6.14–10.30	3.0	8.2	0.9	0.49–0.97	1.5	5.3	1.28–9.22	6.4	− 7.29 to17.29	4.5	12.5
Peak flexion/extension	0.9	0.40–0.97	96.3	3.9	− 2.96–10.82	11.10	− 17.86–25.72	7.8	21.8	0.94	0.74–0.99	96.4	− 0.42	− 4.75–3.91	7.0	− 14.12–13.28	4.9	13.7
Knee joint																		
Peak abduction/adduction	0.92	0.66–0.98	4.9	0.92	− 1.06–2.91	3.2	− 5.35–7.19	2.3	6.3	0.93	0.69–0.98	3.2	2.89	1.02–4.76	3.02	− 3.03–8.81	2.1	5.9
Peak internal/external rotation	0.9	0.10–0.60	3	− 0.64	− 4.35–3.06	5.98	− 12.36–11.08	4.2	11.7	0.87	0.44–0.97	3.3	− 2.17	− 5.43–1.10	5.27	− 12.50–8.16	3.7	10.3
Peak flexion/extension	0.91	0.62–0.98	97.4	− 1.06	− 3.44–1.33	3.85	− 8.60–6.49	2.7	7.5	0.89	0.50–0.97	96.2	− 1.96	− 4.83–0.92	3.02	− 11.05–7.13	2.1	5.9
Ankle joint																		
Peak abduction/adduction	0.96	0.81–0.99	1.7	1.47	− 0.30–3.25	2.86	− 4.13–7.08	2.0	5.6	0.92	0.64–0.98	1	0.21	− 1.32–1.74	2.47	− 4.63–5.04	1.7	4.8
Peak inversion/eversion	0.9	0.54–0.98	16.3	0.17	− 1.11–1.46	2.07	− 3.89–4.24	1.5	4.1	0.92	0.62–0.98	16.5	0.47	− 0.66–1.60	1.82	− 3.10–4.04	1.3	3.6
Peak dorsiflexion/plantarflexion	0.83	0.24–0.96	17.7	− 1.9	− 4.90–1.11	4.85	− 11.39–7.60	3.4	9.5	0.52	− 2	16.7	0.44	− 1.19–2.06	2.62	− 4.70–5.58	1.9	5.1

ICC (intraclass correlation coefficient), 95% CI (the 95% confidence interval for the ICC), Mean (average angle measured between tester 1 on 2 occasions (intra-tester) and tester 1 and 2 (inter-tester)), Mean Diff (represents the average of the differences between two measurements made by tester 1 on two occasions (intra-tester) and between tester 1 and 2 (inter-tester)) and the 95% CI for Mean Diff, SD Diff (the standard deviation of the differences), 95% LOA ( Bland and Altman 95% limits of agreement), SEM (standard error of measurement) and MDC (absolute minimal detectable change)

SEM values were ≤ 5° for intra- and inter-tester trials respectively with the exception of pelvic tilt, rotation, hip flexion/extension and ab/adduction and lumbar flexion/extension (SEM range: 5.1–12.6°, with the largest error in pelvic rotation). Similar to the gait task, mean differences for all parameters between sessions were lower for intra-tester trials (≤ 3.9°) than inter-tester trials (≤ 5.3°). The range of MDC values was wider in the STS task (2.9–34.9° (intra-tester) and 3.6–25.6° (inter-tester)) compared to the gait task with the highest values relating to pelvic rotation in both cases.

Bland Altman 95% limits of agreement for both intra-tester and inter-tester STS trials are outlined in Table 2.

### Discussion

To our knowledge, reliability has not been previously examined amongst experienced and inexperienced testers during both gait and STS tasks using a three rigid segmented spine, pelvic and lower limb model in adults. Similar gait studies, which focussed on a two rigid spine segment model with lower limbs but without pelvic outputs [11, 26], also found small mean intra-tester differences (Mean Diff Intra-tester). The ‘Imperial Spine’ (3 rigid spine segment model including pelvic and lower limb outputs) builds upon this; inter-tester kinematics differences (Mean Diff Inter-tester) were low within both gait and STS tasks.

Systematic reviews of the reliability of 3DMC kinematic measurements have demonstrated that reliability varies between studies due to methodological variation [1, 27], which makes direct comparison difficult. Overall, ICCs are reported to be above 0.7 for most range of movement parameters [1] and are highest within the sagittal plane [27]. These findings concur with this current study (median ICC for gait and STS tasks > 0.89 for intra- and inter-tester data) and that of more recent work [28, 29].

Transverse plane measurements are typically less reliable (median ICC < 0.72) [27]. However, using the ‘Imperial Spine’, the median values are increased in both transverse and frontal planes (median ICC > 0.80 for gait and STS task for intra- and inter-tester data) with the exception of transverse and frontal plane inter-tester ICCs for the STS tasks (median 0.60 and 0.62 respectively). To our knowledge, this has not been investigated until now in healthy adults using a multi-segmental spine and bilateral lower limb model.

In agreement with this current study, higher intra-tester than inter-tester reliability is reported [27] and may represent a difference in tester experience [7]. It is proposed that errors between 2 and 5° are acceptable [27]. In this current study the SEM for STS tasks (intra- and inter-tester) was higher than this, as one would expect for a task requiring through range movement (SEM range 1.0–7.8, except for peak pelvic rotation), and was lower in gait trials (SEM range 0.8–5.5). Although, the corresponding MDC ranges approximate values recently cited during gait and STS tasks [28, 29], the MDC range in our study was wider during STS.

Despite unavoidable and well documented errors implicated in 3DMC, these findings indicate that it should be possible to reliably establish kinematic differences using the ‘Imperial Spine’. In order to identify potential therapeutic targets, further testing will be required in LBP patients.

### Limitations

Although a pragmatic sample size was used in this study [13], reflecting that of previous reliability trials [1, 5, 30], the authors recognise that an increased sample size would have further enhanced reliability and MDC outcomes. Participants were examined by each tester following a 45 min rest period, which could also be considered a limitation. This was necessary to ensure that measurements were made at the same time of day to ensure that the diurnal changes of the spine (disc hydration) in this cohort or changes in movement over time did not account for the changes observed.

It is important to note that the reported error in the ‘Imperial Spine’ relates to healthy participants and therefore, is not applicable to a patient population. Future work will include the examination of spinal, pelvic and lower limb kinematics in LBP patients.

## Supplementary information

**Additional file 1:** **Table S1.** Positioning of the ‘Imperial Spine’ marker set.

**Additional file 2:** **Figure S1.** Mean spine and lower limb sagittal waveforms during gait (left panel) and STS (right panel) tasks.

**Additional file 3:** **Figure S2.** Mean spine and lower limb frontal waveforms during gait (left panel) and STS (right panel) tasks.

**Additional file 4:** **Figure S3.** Mean spine and lower limb transverse waveforms during gait (left panel) and STS (right panel) tasks.

## Data Availability

The datasets used during this study are available upon reasonable request.

## Abbreviations

ASIS: Anterior Superior Iliac Spine
CI: Confidence Interval
3D: Three Dimensional
3DMC: Three Dimensional Motion Capture
ICC: Intraclass Correlation Coefficient
JCS: Joint Co-ordinate System
LFC: Lateral Femoral Condyle
LMAL: Lateral Malleolus
LOA: Level of Agreement
LBP: Low Back Pain
LT: Lower Thoracic
L: Lumbar
Mean Diff: Mean of the Differences
MFC: Medial Femoral Condyle
MMAL: Medial Malleolus
MDC: Minimal Detectable Change
PSIS: Posterior Superior Iliac Spine
SD: Standard Deviation
SD Diff: Standard Deviation of the Differences
SEM: Standard Error of the Mean
STS: Sit to stand
UT: Upper Thoracic
