# Supplementary figures and images for: Reliability and minimal detectable change of the ‘Imperial Spine’ marker set for the evaluation of spinal and lower limb kinematics in adults
Source: BMC Res Notes. 2020 Oct 22;13:495. doi: 10.1186/s13104-020-05295-9 (PMC7579883; doi:10.1186/s13104-020-05295-9)

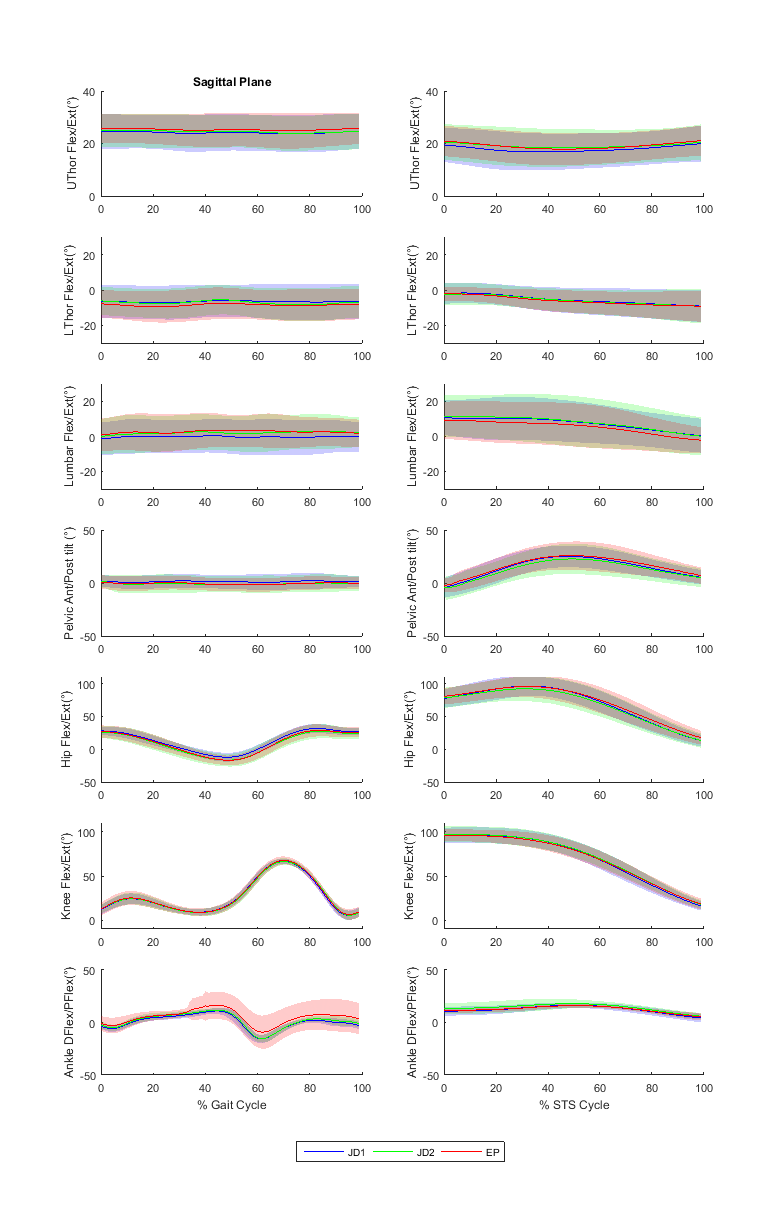

Supplement: Supplementary file 2 — Additional file 2: Figure S1. Mean spine and lower limb sagittal waveforms during gait (left panel) and STS (right panel) tasks. [file 13104_2020_5295_MOESM2_ESM.tiff]

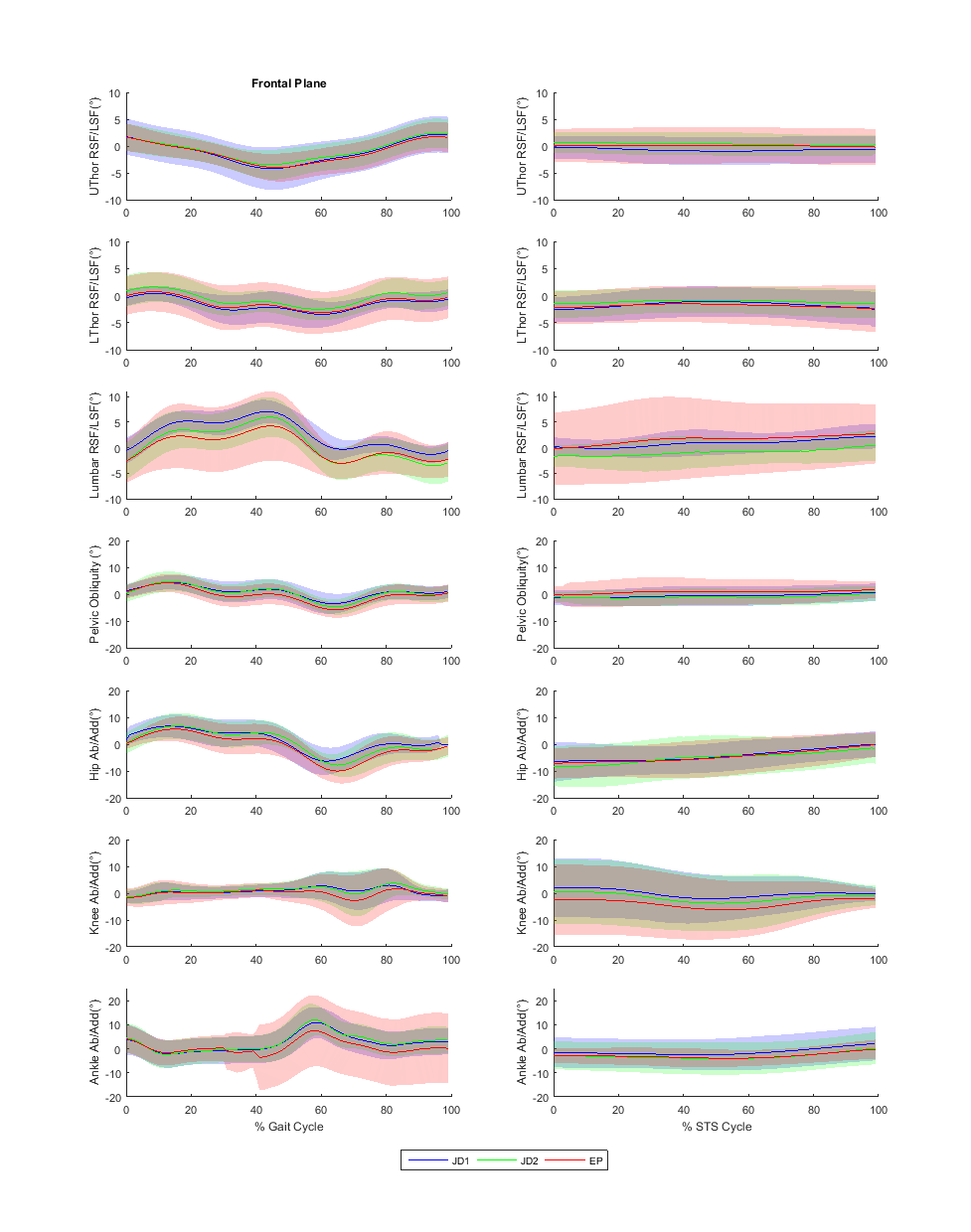

Supplement: Supplementary file 3 — Additional file 3: Figure S2. Mean spine and lower limb frontal waveforms during gait (left panel) and STS (right panel) tasks. [file 13104_2020_5295_MOESM3_ESM.tiff]

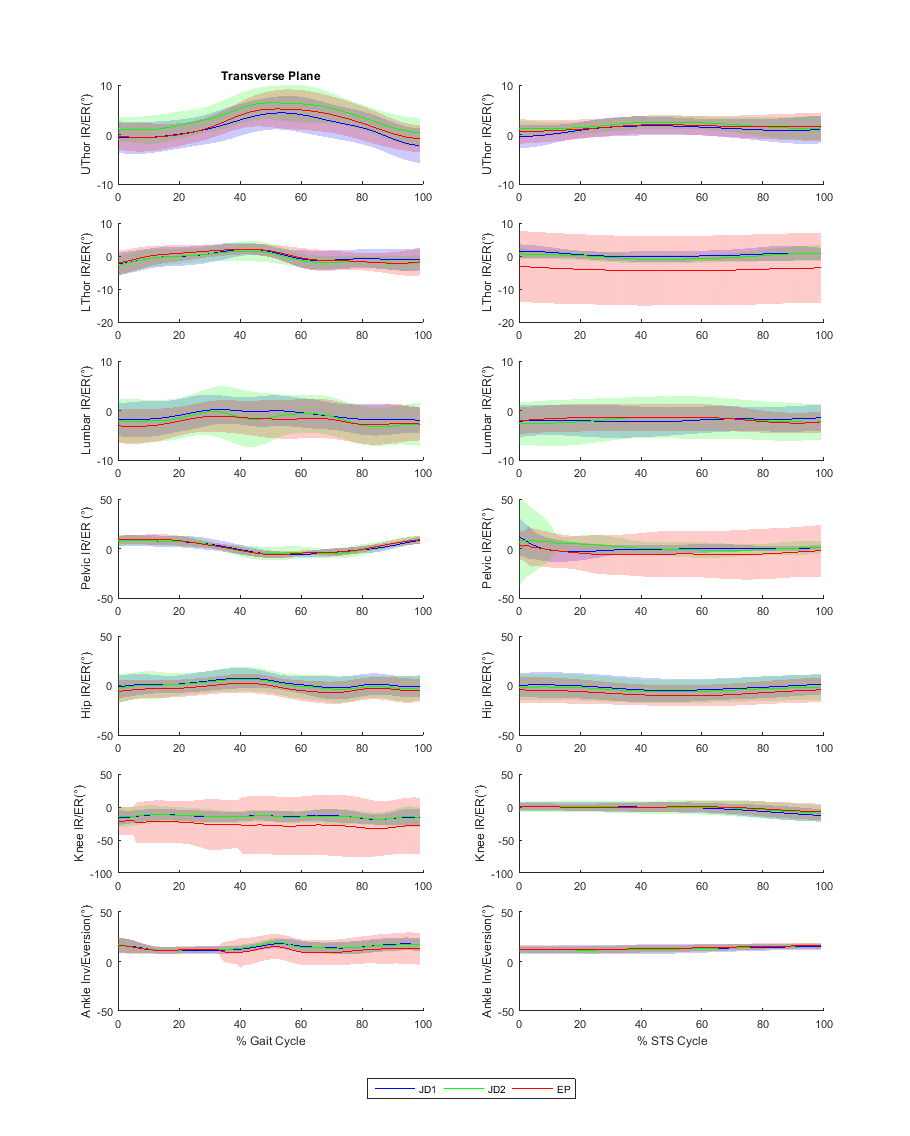

Supplement: Supplementary file 4 — Additional file 4: Figure S3. Mean spine and lower limb transverse waveforms during gait (left panel) and STS (right panel) tasks. [file 13104_2020_5295_MOESM4_ESM.tiff]
